# Supplementary figures and images for: Evolution and lineage dynamics of a transmissible cancer in Tasmanian devils
Source: PLoS Biol. 2020 Nov 24;18(11):e3000926. doi: 10.1371/journal.pbio.3000926 (PMC7685465; doi:10.1371/journal.pbio.3000926)

Figure S1

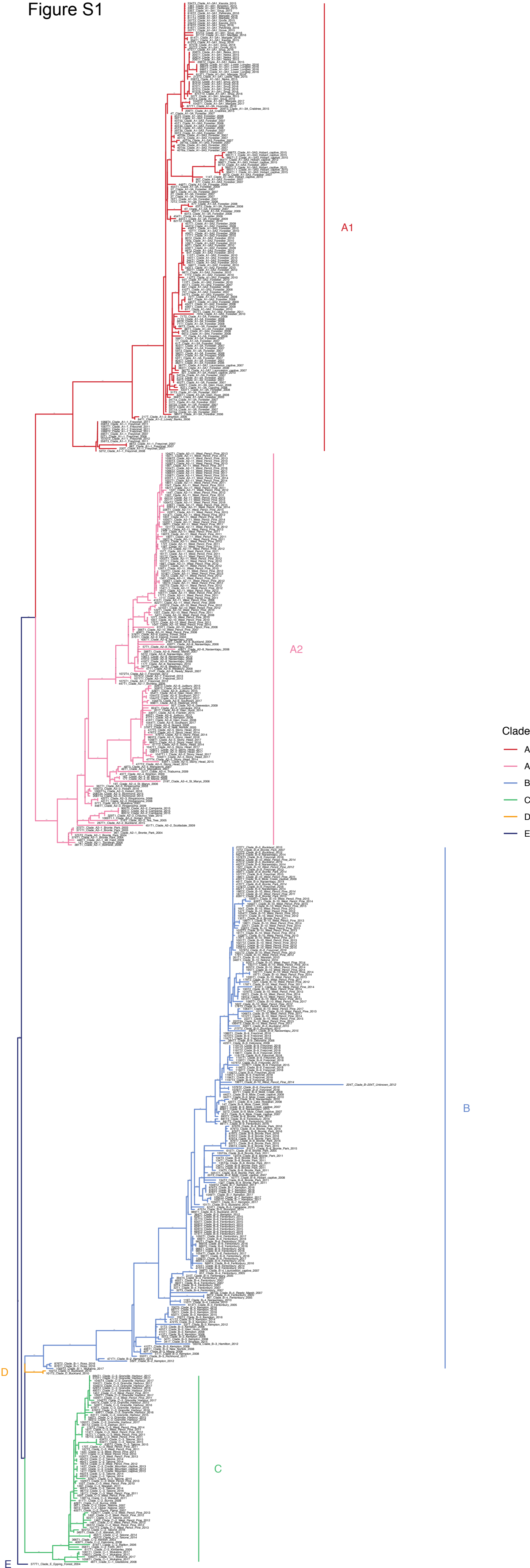

Supplement: S1 Fig — Phylogenetic tree of 639 DFT1 tumours, including cell lines, constructed with 456 MIP genotypes, 94 mtDNA mutations, and 932 CNVs; this is a higher resolution version of the tree shown in Fig 1A. Each tumour is labelled with its ID, location and year of sampling, and clade group (S1 Table). Nine tumours (8T2, 9T, 120T, 166T4, 390T1, 460T1, 478T2, 1070T1, and 1072T1) were excluded due to substantial missing data; these tumours’ clade groups can be found in S1 Table. The 26 tumours labelled in italic font have missing data or very low purity, and their exact positions on the tree may be inaccurate; in these cases, likely true phylogenetic position is presented with the labelled clade group and in S1 Table. Bootstrap values from 1,000 replicates are reported for each node. Some nodes (base of clades A, B, C, and D and that linking samples 332T and 471T1) were constrained, and as a result, bootstrap values are shown as 100. Branch lengths are proportional to number of mutational events, not evolutionary time. Sample information is available in S1 Table, and SNV and CNV genotypes are available in S2 and S3 Tables, respectively. (PDF) [file pbio.3000926.s001.pdf]

Figure S2

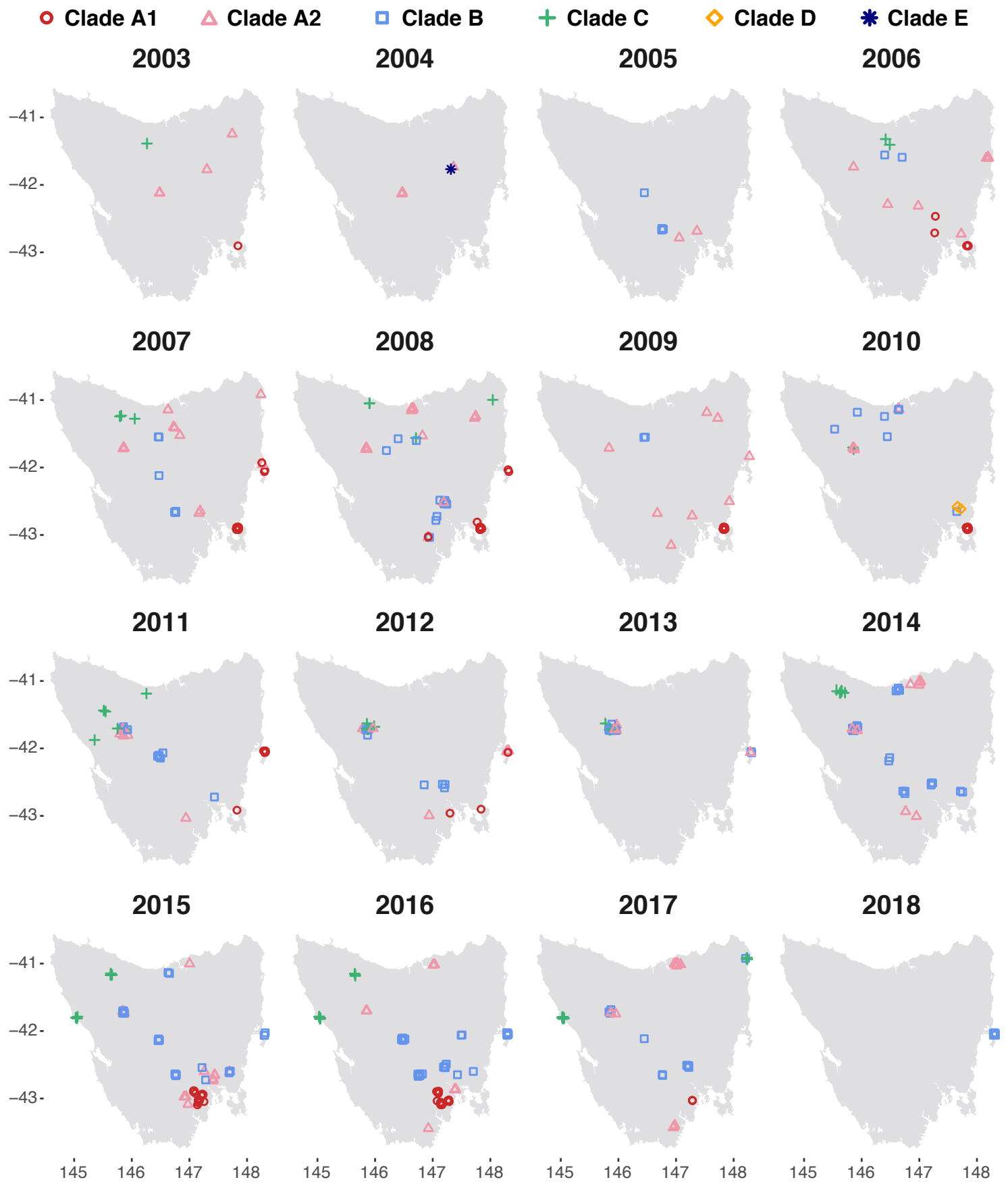

Supplement: S2 Fig — Locations of 593 DFT1 tumours collected between 2003 and 2018, coloured by clade. These are the same data as shown in Fig 2B, but split by year. Map outline was obtained from https://library.unimelb.edu.au/collections/map_collection/map_collection_outline_maps. Underlying data are available in S1 Table. (PDF) [file pbio.3000926.s002.pdf]

Figure S3

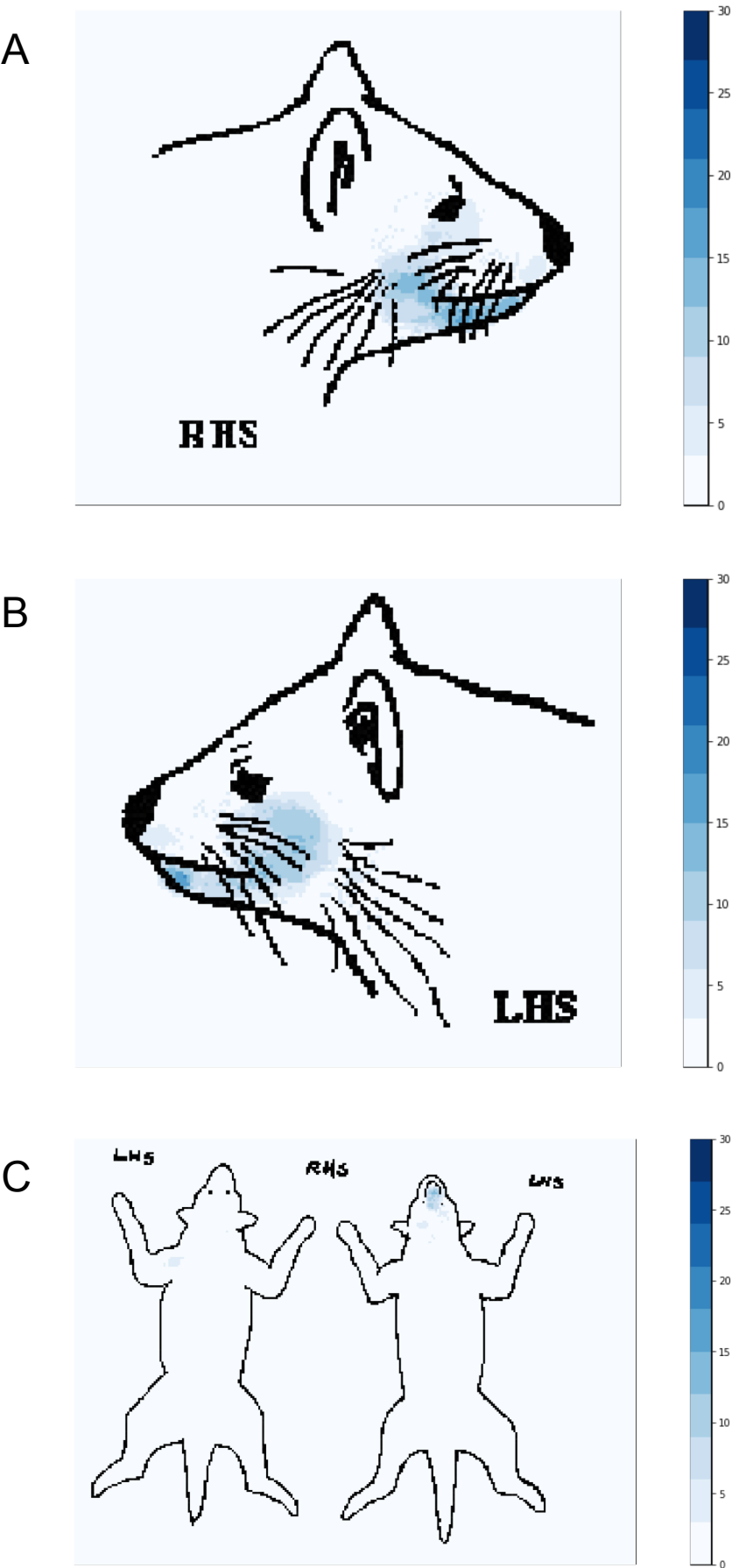

Supplement: S3 Fig — Locations of confirmed DFT1 tumours illustrated on Tasmanian devil (A) face, right-hand side, (B) face, left hand side, and (C) body diagrams. Scale represents number of tumours. The number of individuals in the sample with tumours within the relevant body area were 36, 32, and 32, top to bottom. Underlying data are available in S1 Data in https://doi.org/10.5281/zenodo.4046235. (PDF) [file pbio.3000926.s003.pdf]

Figure S4

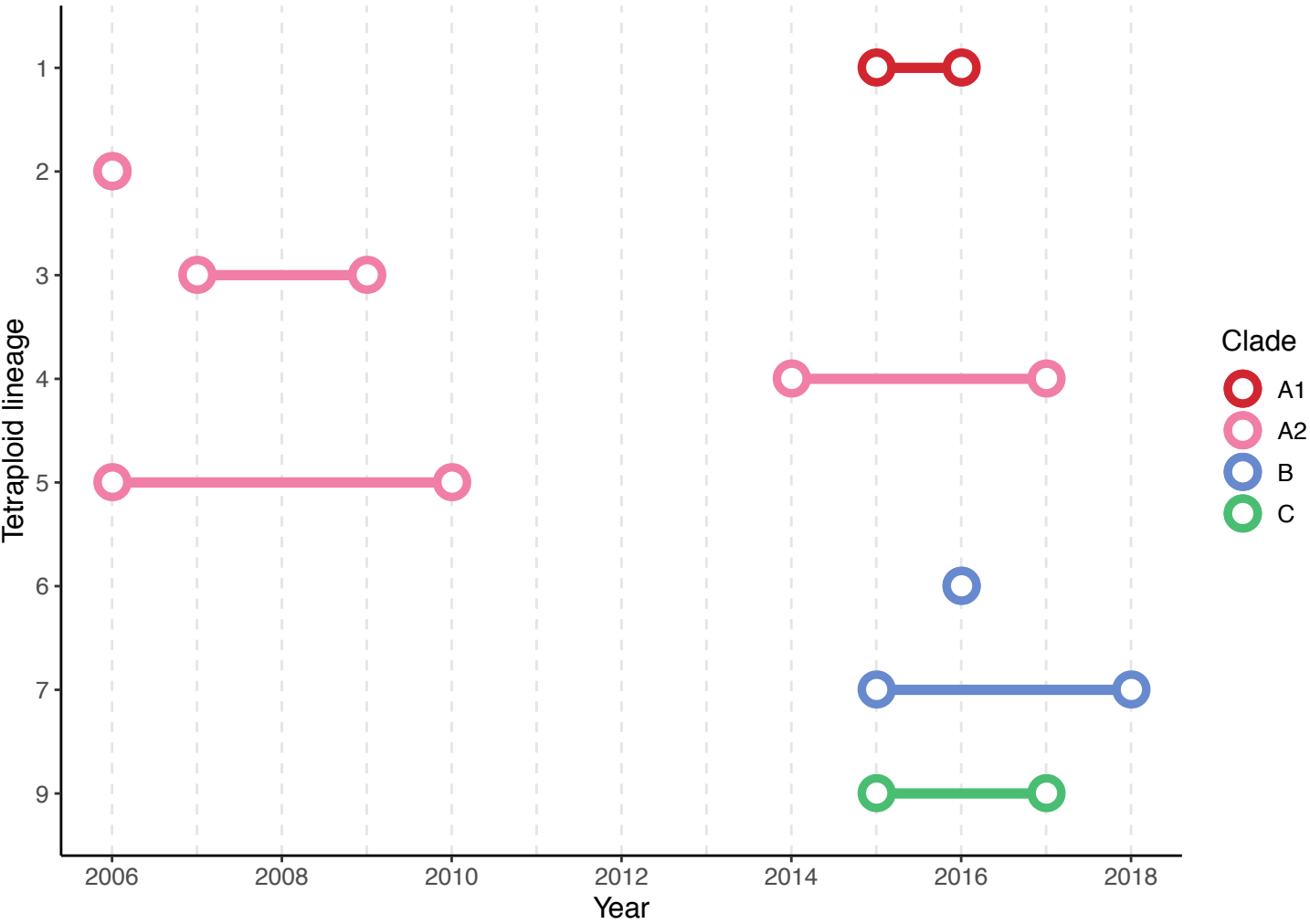

Supplement: S4 Fig — Clade and temporal distributions of the eight observed DFT1 biopsy tetraploid lineages. The years of first and most recent observations of each lineage are indicated. Each lineage is labelled with a numeric ID. Lineage 8 was observed only in a cell line and is not included. Data are available in S1 Table. (PDF) [file pbio.3000926.s004.pdf]

Figure S5

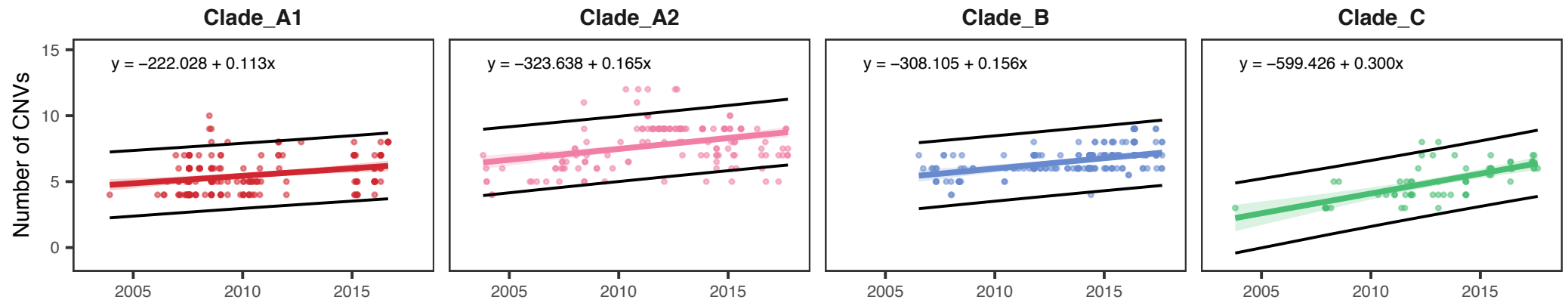

Supplement: S5 Fig — Rate of CNV acquisition in DFT1 clades. X-axis represents collection date, with labels representing 1 January of the labelled year. Sets of CNVs which cooccur with the same copy number in the same samples are counted once, and we corrected for ploidy in tetraploid tumours. DFT1 cell lines were excluded. The difference in rate between clade C and the other clades is statistically significant (p < 0.005, pairwise contrasts of emmeans, with Tukey multiple testing adjustment). Coloured shading represents regression standard error, and upper and lower lines represent prediction intervals. Data are available in S1 Table (Total_CNVs_linked.clonal_copynumbers_only) and S3 Table. (PDF) [file pbio.3000926.s005.pdf]

Figure S6

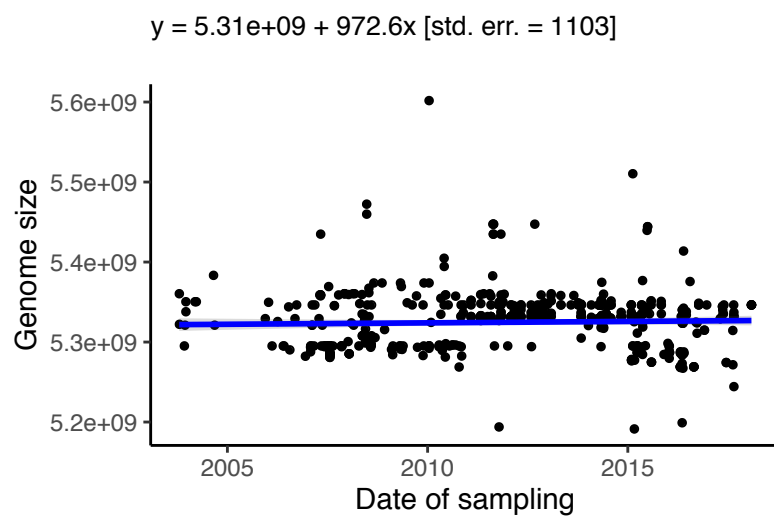

Supplement: S6 Fig — Diploid genome size was estimated for each tumour by adding and subtracting CNV gain and loss footprints from the diploid Tasmanian devil callable genome size (haploid size, 2,625,758,988 bp). X-axis represents collection date, with labels representing 1 January of the labelled year. Subclonal CNVs, tetraploid tumours, duplicate tumours, and cell lines were excluded. Each tumour is represented by a dot, and shading represents regression standard error. Robust regression was performed, p-value for association = 0.3849 (robust F-test). Data are available in S1 and S3 Tables. (PDF) [file pbio.3000926.s006.pdf]

Figure S7

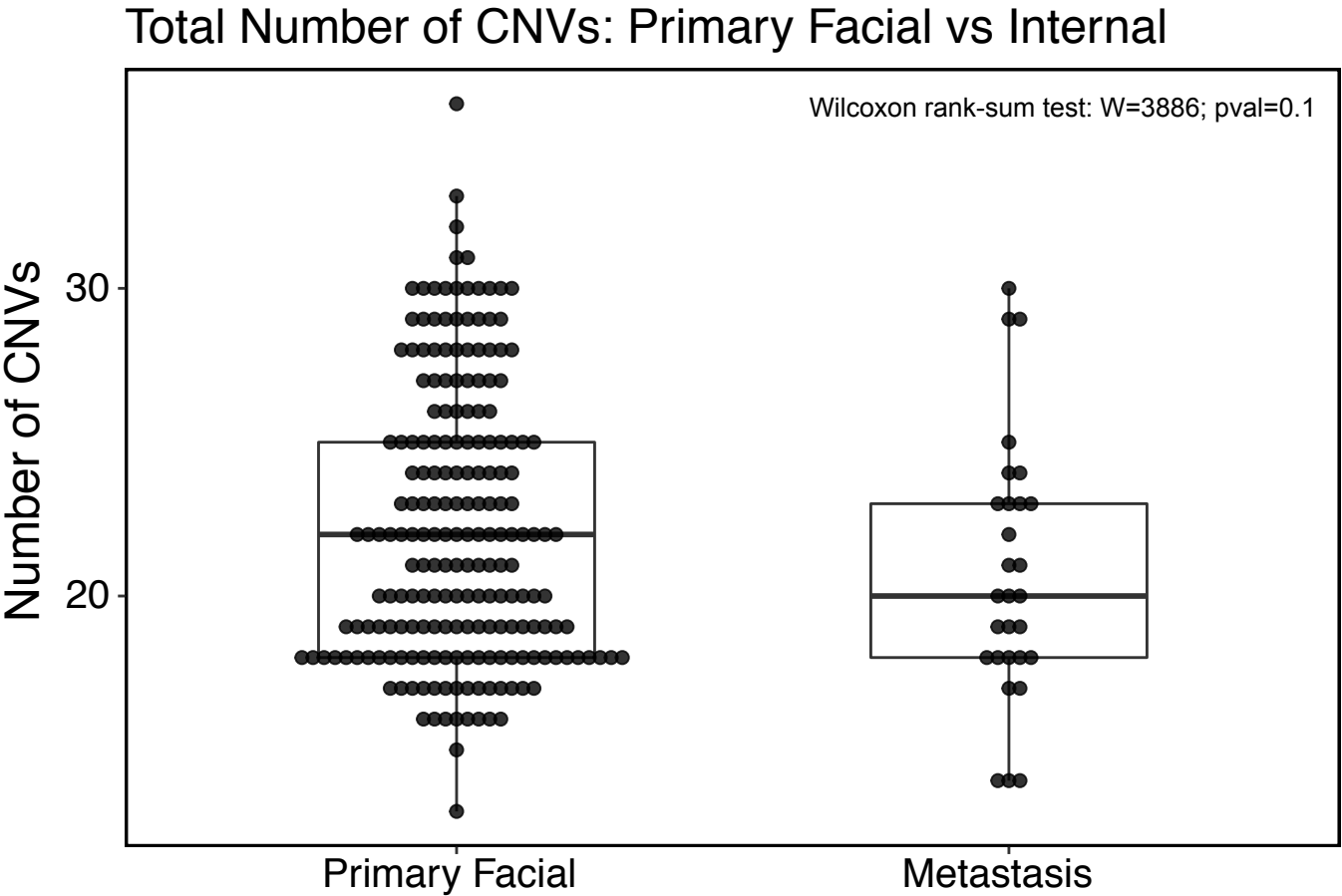

Supplement: S7 Fig — Number of CNVs does not differ between DFT1 biopsies collected from primary facial tumours (n = 543) and internal metastases (n = 24). Each dot represents a tumour. Data are available in S1 Table. (PDF) [file pbio.3000926.s007.pdf]

Figure S8

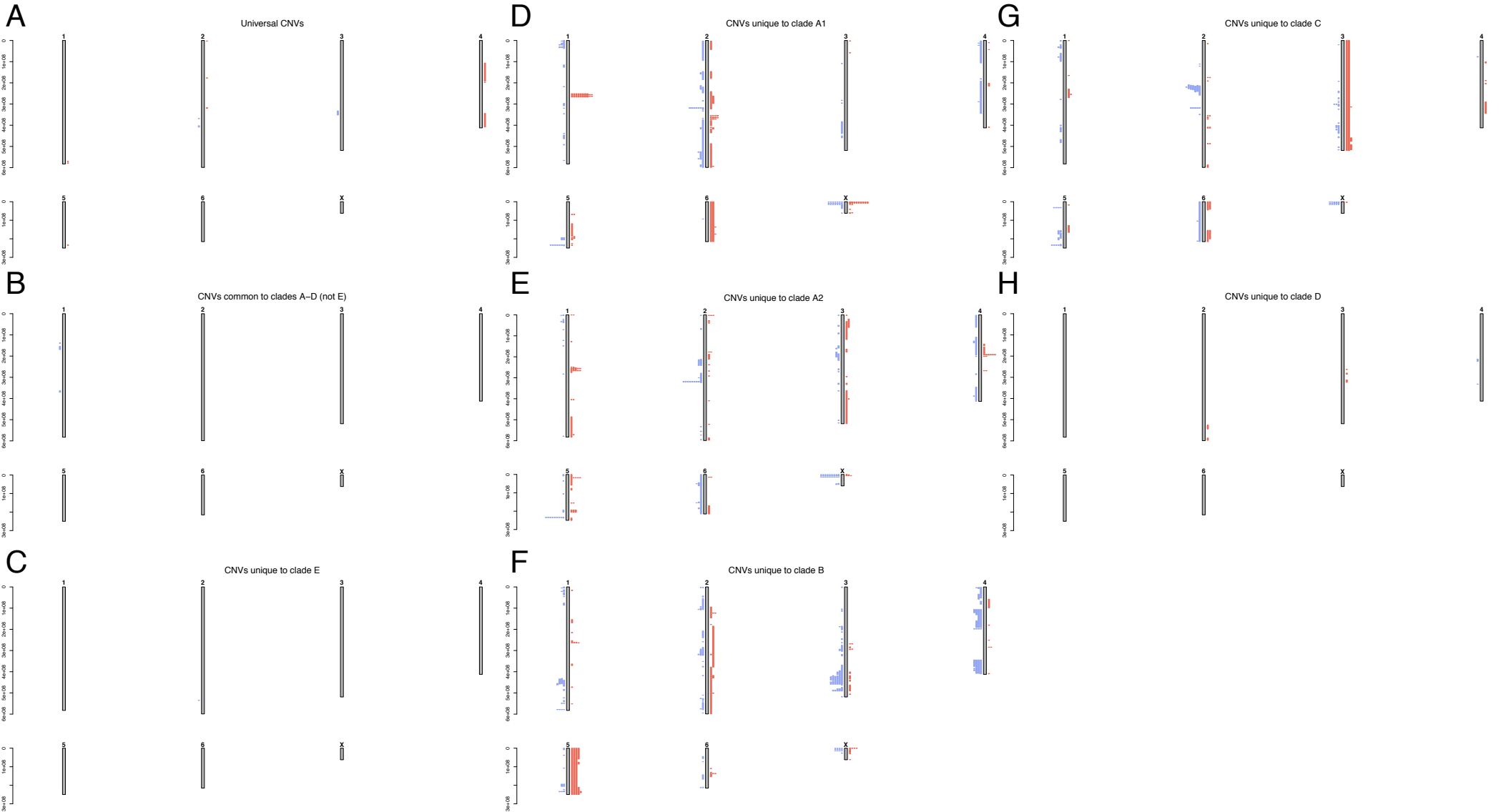

Supplement: S8 Fig — Chromosome map illustrating subsets of CNVs in DFT1 biopsies (A) shared by all DFT1s; (B) exclusive to and shared by all DFT1s in clades A, B, C, and D; (C) exclusive to clade E; (D) exclusive to clade A1; (E) exclusive to clade A2; (F) exclusive to clade B; (G) exclusive to clade C; (H) exclusive to clade D. Grey bars represent chromosomes, with scale on left in bp. Blue and red bars represent copy number losses and gains, respectively. Each bar is an independent CNV occurrence, and CNVs shared by multiple tumours via a common ancestor are illustrated once. Each CNV step away from CN2 is illustrated separately, thus a CNV present at CN <1 or >3 is shown multiple times. Data are available in S3 Table. (PDF) [file pbio.3000926.s008.pdf]

Figure S9A

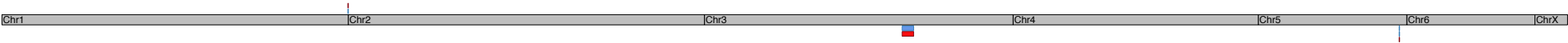

Figure S9B

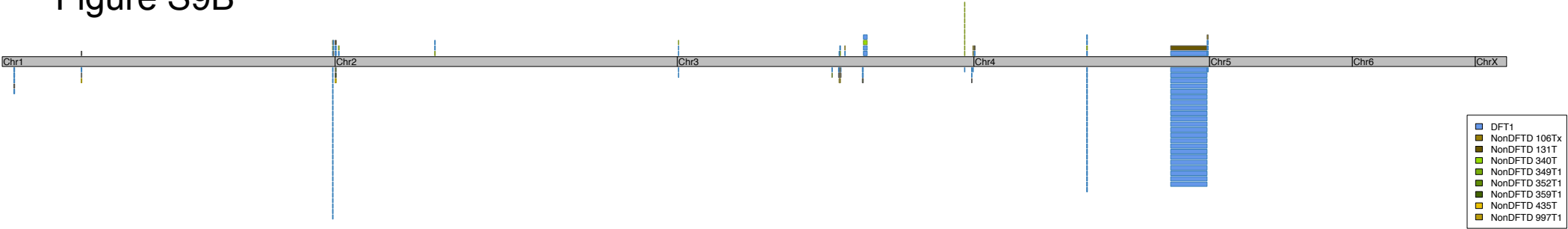

Figure S9C

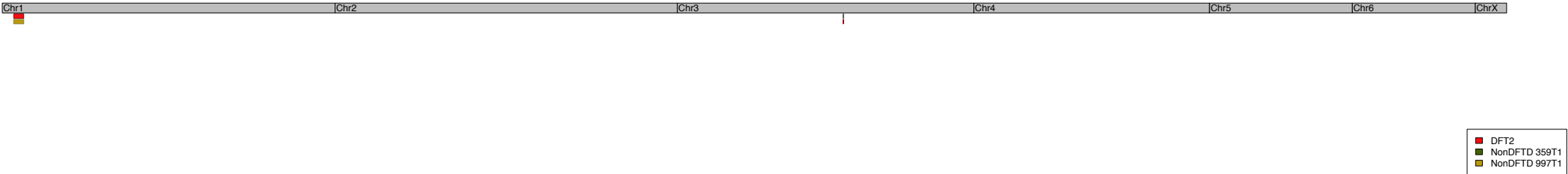

Supplement: S9 Fig — Chromosome map illustrating recurrent CNVs with identical breakpoints (at 100-kb resolution) occurring within one or more tumours belonging to each cancer group in each of the subsets: (A) DFT1 and DFT2; (B) DFT1 and non-DFTD; and (C) DFT2 and non-DFTD. Non-DFTD, non-devil facial tumour disease, i.e., neither DFT1 nor DFT2. Grey bars represent chromosomes, and gains and losses are represented by coloured bars above and below the chromosomes, respectively. The colour key identifies the tumours whose CNVs are represented by each colour. Three non-DFTD tumours (106T1, 106T2, and 106T3) belong to the same non-DFTD cancer sampled from 3 body sites and CNVs were collapsed (106Tx). No CNVs occurring independently in more than one non-DFTD tumour, or in one or more DFT1, DFT2 and non-DFTD tumour were found. This figure refers to recurrent CNVs between devil cancers, and differs from Fig 2I, which describes reused single breakpoints. Underlying data are available in S3 Table. (PDF) [file pbio.3000926.s009.pdf]

Figure S10

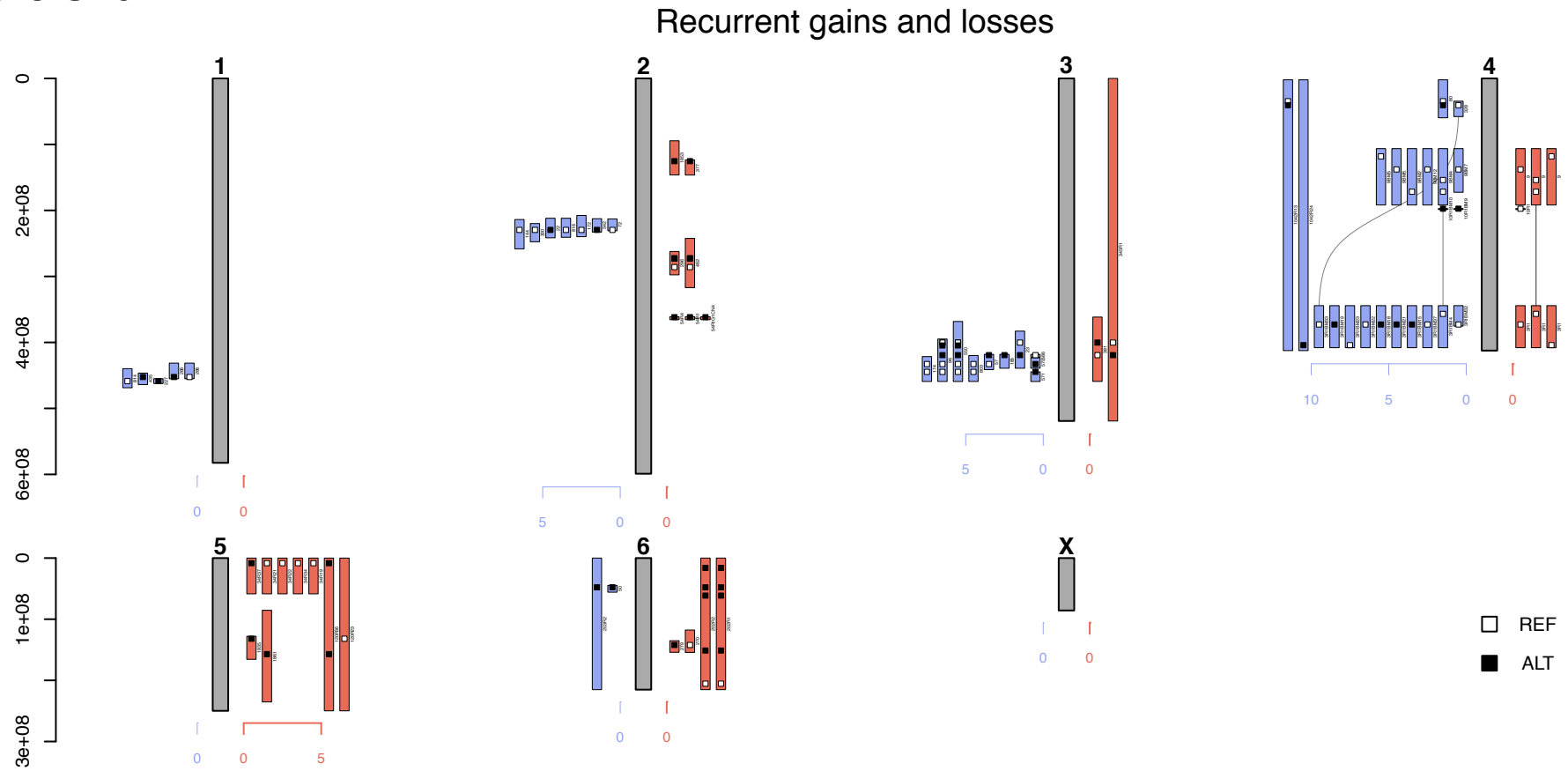

Supplement: S10 Fig — CNV chromosome map displaying CNVs from DFT1 biopsies and cell lines whose footprint encompassed genotyped MIP alleles (S2 Table and S3 Table). MIP genotypes and CNVs are only shown if the CNV depth in a single direction (gain, loss) was ≥2 and if the MIP ALT allele was either present in the tumour(s) carrying the CNV (gains) or inferred to be present in an ancestor tumour (losses). Gains are coloured red and losses blue, and chromosome coordinates in bp are shown in scale bar on left. The allele (REF, ALT) implicated in each CNV is shown and CNV IDs (S3 Table) are labelled. CNVs 3R1 and 9, parental CNVs of the recurrent chromosome 4 back mutations observed in cell lines, are shown three times each in order to illustrate informative MIPs genotypes that were captured in different samples. The figure shows only directly observed genotypes, although in some cases additional genotypes can be imputed. Lines connect CNVs that cooccur on the same chromosome in the same direction (gain, loss) in the same tumour(s) and which may be linked. None of the stacked genotyped CNVs reached the minimum threshold (9) needed to obtain sufficient power to detect allelic skew with a Fisher exact test. Data are available in S11 Table. (PDF) [file pbio.3000926.s010.pdf]

Figure S11

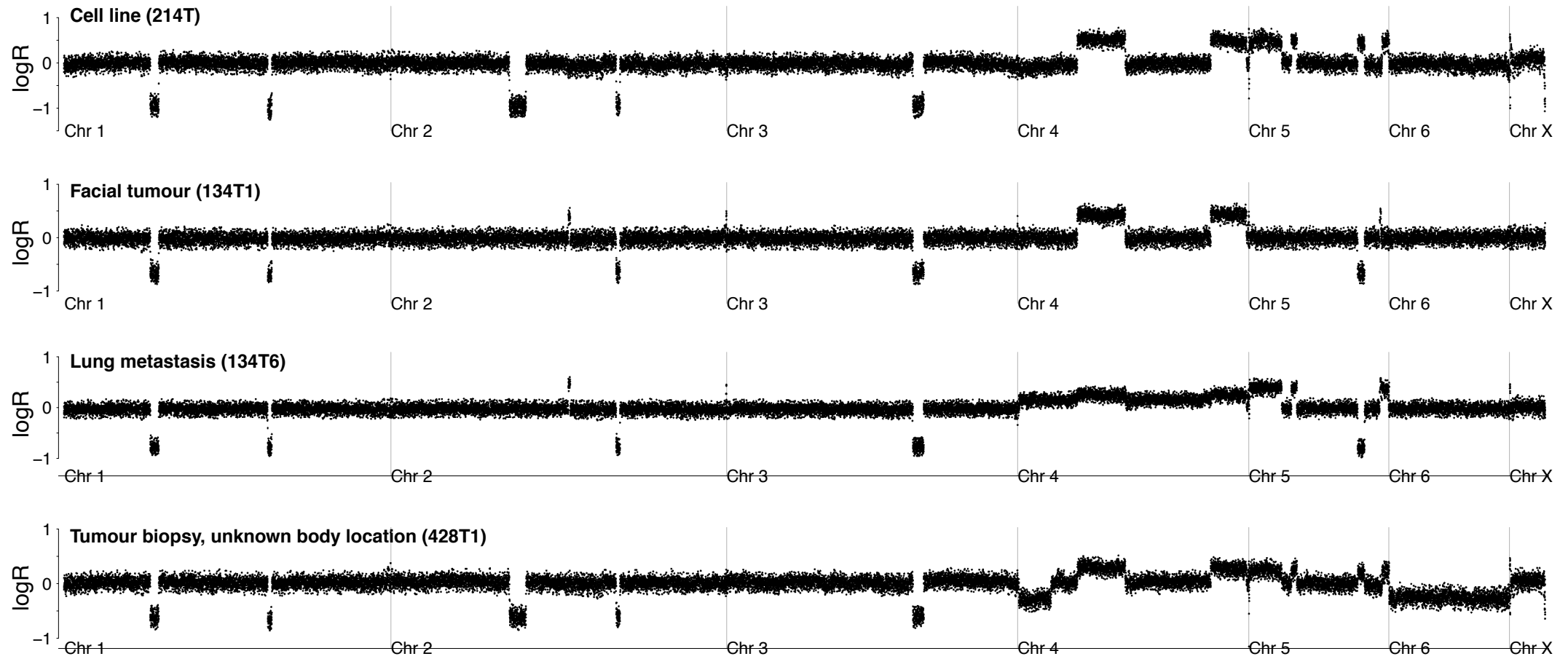

Supplement: S11 Fig — Genomic copy number plots for two DFT1 tumours, a lung metastasis (134T6 and a tumour biopsy of unknown body location (likely facial) (428T1)), with CNV profiles similar to those seen in DFT1 cell lines. The changes observed in 428T1 occurred after whole genome duplication. Copy number plots from the primary facial tumour from the same animal as the lung metastasis (134T1), and a representative cell line (214T), are also displayed. Each dot represents normalised read coverage within a 100-kb genomic window. R, ratio of tumour to normal reads, log base 2. Tumour identifiers are labelled in parentheses. Copy number plots for all tumours are available in S2 Data in https://doi.org/10.5281/zenodo.4046235. (PDF) [file pbio.3000926.s011.pdf]
