# Supplementary material for: Evolution and lineage dynamics of a transmissible cancer in Tasmanian devils
Source: PLoS Biol. 2020 Nov 24;18(11):e3000926. doi: 10.1371/journal.pbio.3000926 (PMC7685465; doi:10.1371/journal.pbio.3000926)
Supplement: S12 Fig — (A) Telomeric repeat length in DFT1, DFT2, and normal devils (“hosts”). Each sample is represented by a dot. Five outliers in the “host” group are not shown on the plot but were included in the data used to generate boxplot. Data are available in S1 Table. (B) Linear regression of telomere repeat length and year of collection for DFT1 biopsies with tumour purity included as a covariate. Residual errors are plotted in red, and blue shading represents the standard error of the gradient estimate. In both (A) and (B), telomere length is the cumulative length of telomere repeat (TTAGGG) detected in the genome, including both telomeric and interstitial repeats, which cannot be distinguished using this method. (PDF) [file pbio.3000926.s012.pdf]

Figure S12

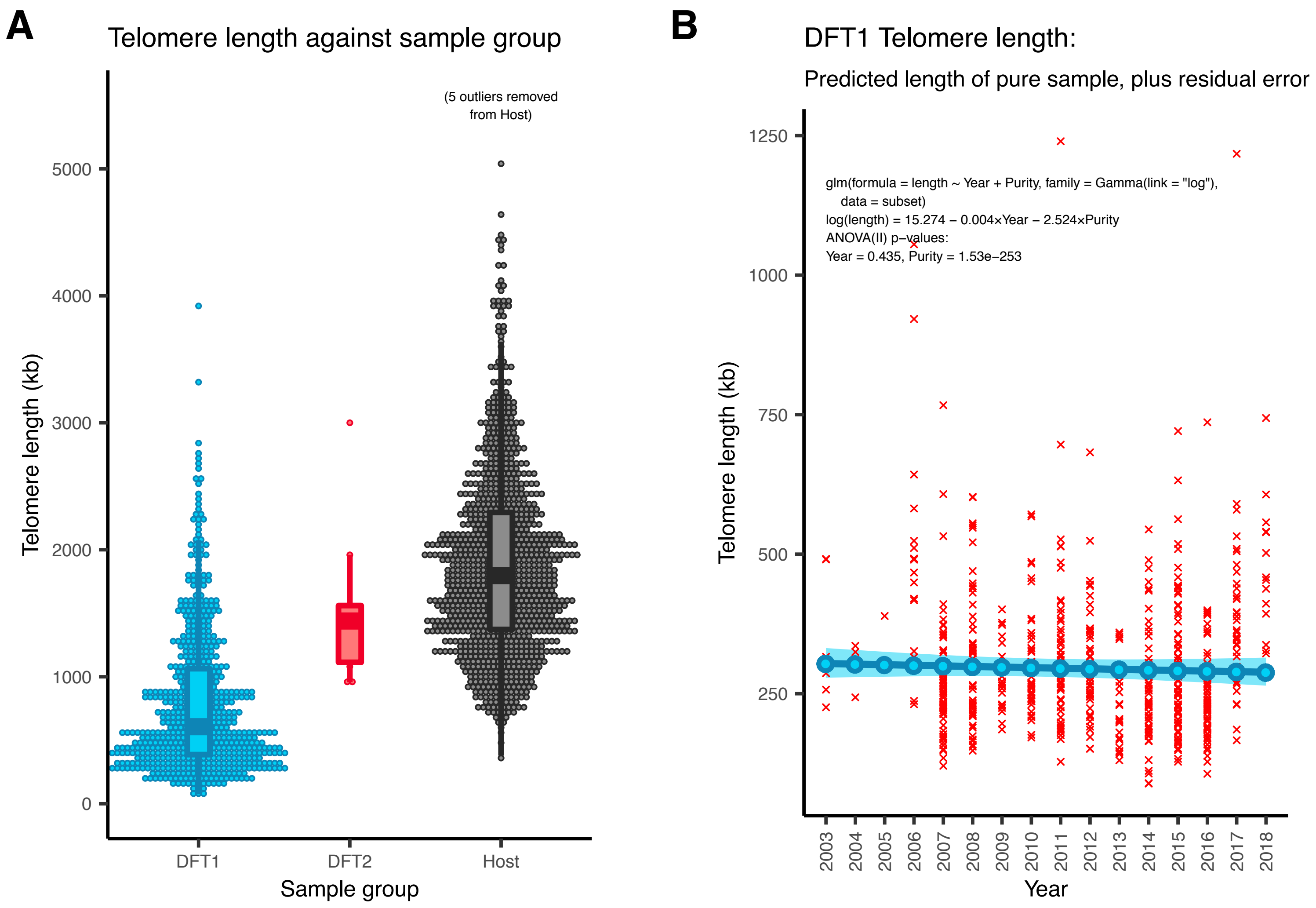
A
Telomere length against sample group
(5 outliers removed from Host)
Telomere length (kb)
0
1000
2000
3000
4000
5000
DFT1
DFT2
Host
Sample group
B
DFT1 Telomere length:
Predicted length of pure sample, plus residual error
glm(formula = length ~ Year + Purity, family = Gamma(link = "log"),
data = subset)
log(length) = 15.274 − 0.004×Year − 2.524×Purity
ANOVA(II) p−values:
Year = 0.435, Purity = 1.53e−253
Telomere length (kb)
250
500
750
1000
1250
2003
2004
2005
2006
2007
2008
2009
2010
2011
2012
2013
2014
2015
2016
2017
2018
Year
