# Supplementary material for: Evolution and lineage dynamics of a transmissible cancer in Tasmanian devils
Source: PLoS Biol. 2020 Nov 24;18(11):e3000926. doi: 10.1371/journal.pbio.3000926 (PMC7685465; doi:10.1371/journal.pbio.3000926)
Supplement: Attachment — Submitted filename: Kwon_ResponseToReviewers.pdf [file pbio.3000926.s025.pdf]

Reviewer #1:

Mi Kwon et al. is a very interesting study of the unique transmissible cancer in Tasmanian devils. The paper has two main parts: The first uses genomic data to show the evolutionary history of DFT1 and how the clades have spread across the population and replaced each other in different populations multiple times. The second analyzes the copy number changes over time in order to better understand the genetic stability of transmissible cancer. This study analyzed a massive number of DFT1 samples and provides a significant increase in our knowledge of DFT1 evolution at both the ecological level and the level of genetic and chromosomal stability. It will be of broad interest to researchers interested in biology, cancer, ecology, and pathogens. I have several minor questions and comments below.

1. Page 4, line 4: "Remarkably, of the one hundred devils hosting two or more DFT1 tumours within the cohort, 37 had tumours with 'public' genotypes shared between tumours in more than one individual (Figure 1E and Table S4). Most of these likely represent co-infection, but in some cases we may have sampled the source of a new genotype." Is this exactly 100 or about 100? The exact number should be here, and I just want to double check as 100 is a very round number.
This does indeed happen to be exactly 100. We have included the word “exactly” in the text in order to prevent confusion on this point.

Also, the number of total cases considered here should be shown in the text and/or figure legend (100 out of how many?) When making a conclusion about the rate of coinfection it could very easily be misinterpreted as saying that 37% of cases are co-infection. The actual rate of coinfection is important and relevant in many real ways and it should be stated, while the % of devils carrying two tumours that are co-infected is not.
A note has been added to the legend for Figure 1E, stating that 277 devils had only a single tumour sampled. These data are also available in Table S4.

The description of "public" genotypes is confusing here, and could be clarified. This is explained well in the figure legend. Perhaps the two sentences in the figure legend could be moved to the main text.
We have altered the wording of this section in order to improve clarity.

2. Page 4, line 26 and Figure 1, I would love to know where the tetraploid cancers fit within this evolutionary tree and geographic spread! The data in Figure S4 show the presence of the tetraploids over time, but do not show their prevalence. Is there any evidence that tetraploid subclades were successfully able to replace diploid strains or were they short-lived in all cases? Due to the possible role of polyploidy in human cancer it would be of interest to know whether tetraploidy in DFT1 was ever able to out-compete diploid cancers or whether it was less successful in all cases.
Tetraploid tumours are labelled in Table S1, so readers can access the information that the reviewer requested. We would also love to know the evolutionary consequences of whole genome duplication for DFT1. However, given that our sampling was opportunistic and inconsistent, we unfortunately cannot make a definitive statement on this topic.

3. Figure 2A. How are tetraploids dealt with in this analysis? A line about this could be added to the legend.
CNVs occurring in tetraploid tumours were excluded from Figure 2A. This has been clarified with a sentence in the figure legend.

4. Page 4 line 28 and Figure 2B, What are the A-E and A-D ancestor genotypes? In the text you describe the creation of a single ancestor. It appears you have made 2 ancestors, one an ancestor of clade A and E, and one from clade A and D. The A-E clade is clearly earlier and contains all DFT1 samples, and has an interesting lack of the loss at B2M. The A-D ancestor is unclear. Due to the ambiguous placement of D in the tree, it is unclear whether this ancestor includes clade B or C or both. If it includes all DFT1 samples except E that would be useful to state.
We apologise for this confusing notation. The clade A-D common ancestor indeed represents the common ancestor of clades A, B, C and D. This has been clarified in Figure 2B and its legend.

5. Page 4, line 37. The text refers to Figure 2B lower panel as an example of a cancer with a high CNV burden, but that panel appears to show a tetraploid cancer with minor additional changes compared to the diploid example. Was this a mistake? Was there an additional example that was supposed to be here?
This is not a mistake. The tumour shown in Figure 2B, lower panel, is indeed an example of a tumour with a particularly high CNV burden. The number of CNVs per tumour can be found in Table S1.

6. Figure 2C. Are these CNVs relative to the normal devil reference or relative to a DFT ancestor?
These CNVs are called relative to the reference genome. This has now been noted in the Figure 2C legend.

7. Page 5, line 15. The number of occurrences of a gain or loss is not necessarily evidence of the selection of cancers with these events. Were these independent events found in a single animal or was there evidence of their spread? This could be critical evidence of selection for these events. For example, M5 has been lost multiple times, but the authors do not consider it to be positively selected—this is hypothesized to be an unstable locus with no selection for or against. The finding of unstable recurrent sites of mutation in Figure 2I also argue that this may not be due to selection. I think selection is an interesting question and might be addressable with the data here, but it has not been shown.
We thank the reviewer for this interesting comment. We are unable to directly distinguish between recurrent CNVs driven by selection and those which have arisen due to neutral processes. The loci which we considered the best candidates for selection were those which (1) have independently undergone copy number changes in the same direction (i.e. gain, loss) on three or more occasions; (2) involve focal regions (<50 Mb); (3) involve CNVs with offset rather than identical breakpoints; (4) include at least one sample with high amplitude copy number change (i.e. copy number <1 or >3); and (5) contain genes with known roles in cancer whose pattern of mutation in human cancer is consistent with the pattern observed in our data (i.e. we would expect to see biallelic inactivation of a recessively-acting tumour

suppressor gene and copy number gain of a dominantly-acting oncogene). We believe that the repeated loss of marker 5 is unlikely to be driven by selection as its loss returns the majority of its sequence to a diploid state, and it is difficult to conceive how the few known cancer genes encoded on marker 5 could drive a selective advantage through a transition from copy number three to two. These ideas are outlined in the "DFT1 copy number variation" section of the main text.

Also, how was the determination made that a gain or loss was independent? Was this based on determination of the breakpoint junction or based on phylogenetic analysis?
This was based on phylogenetic analysis, as outlined in the "Copy number assignment" section of the Methods.

8. Figure 2F. What are the statistical tests comparing? In particular, is it testing whether the M5 losses in clade B are lower than expected, or that clade A2 are higher, or that overall the patterns of predicted and observed are different? And expected based on what? Presumably expected based on the total number of losses observed and the frequency of observation of the ploidy/clade? This could be succinctly mentioned in text or legend.
The two Fisher's exact tests presented in Figure 2F compare the overall observed distributions with those expected by chance. The number of M5 losses expected by chance was calculated assuming equal opportunity for M5 loss amongst all M5-positive tumours, correcting for ploidy. The data and calculations are laid out in Table S8C. This information has now been added to the Figure 2F legend.

9. Figure 2H. Are the number of CNVs for DFT1 and DFT2 samples comparing each DFT sample with the reference or identifying unique CNVs for each sample or relative to the DFT ancestors? It seems like they are total CNVs relative to the reference, but that is suggesting that a primary carcinoma in a single devil has more CNVs than DFT1 has in its total >20 year history.
The reviewer's interpretation is correct. The number of CNVs is relative to the reference genome, and, over its >20 year history, the number of CNVs observed in individual DFT1 tumours is indeed significantly lower than the number observed in individual devil carcinomas. This has now been clarified in the Figure 2H legend.

10. Page 6, line 22. This analysis is unclear. "One hundred and nineteen CNV breakpoints (at 100 kb resolution) were reused across two or more independent devil cancers" Does this refer to the non-DFT cancers? Or is this including non-DFT cancers and DFT1 and 2? Or "independent" DFT samples? I think the first, but it is not clear.
DFT1 and DFT2 are each considered independent devil cancers, and each non-DFT cancer is considered an independent cancer. The five combinations of between-cancer breakpoint reuse are shown in Figure 2I. A comment has been added to the text and the Figure 2I colour key has been reworded in order to clarify this point.

In Fig 2I it appears that the first two bars a samples DFT1 and DFT2 respectively, but maybe they instead represent simulated and observed data from a DFT1/DFT2 comparison. But the column for DFT1 in the DFT1/non-DFTD dataset appears to have blue in it, so it is not just simulated data, so I cannot quite figure it out.

We apologise for the difficulty interpreting Figure 2I. Indeed, the first diagonally striped bar of each pair represents purely simulated data, whereas the second bar represents the real data. Both real and simulated data are further split into reuse subcategories as outlined in the colour key. We have now added slashes to the X-axis category labels to improve clarity, and have added a note clarifying the colour key in the figure legend.

Additionally, since there are so many independent cases of CNV at the same sites in independent cancers, have the authors considered that the CNV might be a germline polymorphism in the population, rather than a recurrent somatic mutation.
We did not find the reused breakpoints presented in Figure 2I in the genomes of normal devil tissues, including those of matched hosts, suggesting that they are likely to be somatic. A note has been added to the Figure 2I legend to clarify this point and normal devil copy number plots have now been made available as S3 Data.

11. Figure S9. In the legend the authors state: "No CNVs occurring independently in more than one non-DFTD tumour, or in one or more DFT1, DFT2 and non-DFTD tumour were found." This statement occurs in the legend of the supplementary figure that is describing the recurrent CNVs with identical breakpoints. Figure 2G and 2I both appear to show CNVs occurring independently. That was a major conclusion of that section in the paper. Is there a typo in this sentence or am I totally misunderstanding the authors' statements? This needs to be clarified.
Figure S9 refers to reused CNVs (i.e. a pair of reused breakpoints leading to recurrent gain or loss of a DNA segment), whereas Figure 2G and 2I refer to reused breakpoints (i.e. a single genomic coordinate defining the start or end of a CNV). A note has been added to the Figure S9 legend in order to emphasise this point.

12. Page 6, line 30, "Laboratory cell lines can be readily established from DFT1 tumours." Is there a reference for this?
Two references have now been added.

13. Page 6, line 36, "Altogether, this suggests that introduction to cell culture may select for genetically unstable DFT1 subclones." Rather than selection for a greater rate of mutation, it seems like an equally likely hypothesis that there has been a stable rate of mutation and relaxation of negative selection for maintenance of genes required for growth and spread in the host. The genes not needed for survival in a dish, but needed for survival in a host would be a very interesting question to pursue in the future.
We have added a comment to the main text acknowledging this possibility.

14. Also, what was the outcome of the 9 cultured DFT lines that were inoculated into live devils? How long did the cells grow before they were screened for the presence of the CNVs? These details should be included somewhere.
The experimental inoculations were part of an immunotherapy trial and have been described elsewhere (Tovar et al Sci Rep. 2017;7:43827), and this paper is cited in our manuscript. Where available, data on the inoculation dates have been added to Table S1.

We thank the reviewer for their useful and constructive comments.

Reviewer #2:

Young et al present a very interesting study on the evolution and spread of a transmissible tumour in Tasmanian devils. This unusual tumour provides a fascinating system in which to study tumour evolution and the authors have done an excellent job of uncovering how the tumour has changes as the disease spread across Tasmania. The paper is well written and the extensive analyses performed conveniently displayed in an easy to understand yet comprehensive figures. I commend the authors on making their findings so accessible for a broad audience. I also commend the authors on the inclusion of an examination of tumour evolution under cell culture conditions vs tumour biopsies. Overall, this is an impressive study. I only have one minor comment that the authors many wish to address:

When I looked at Table S1, I noticed that there was information for a portion of the samples on the tumour strain. I think it would be useful to point out that the clades identified in this study do not match the karyotypic strains identified in Pearse et al 2012 and why this might be. The presence/absence of M5 was used as a point of difference between strains 1 and 2 but the results present from the sequence analyses indicates that M5 has been lost multiple times and isn't indicative of a different tumour "strain".

We are glad that the reviewer found the study interesting, and we thank them for their suggestion. We have now included a comment in the main text on the interpretation of karyotypic strains.
